# Leonurine promotes the maturation of healthy donors and multiple myeloma patients derived-dendritic cells *via* the regulation on arachidonic acid metabolism

**DOI:** 10.3389/fphar.2023.1104403

**Published:** 2023-01-23

**Authors:** Cheng Chen, Lin He, Xi Wang, Rong Xiao, Shu Chen, Zichen Ye, Xuemei Wang, Yu Wang, Yizhun Zhu, Jingying Dai

**Affiliations:** ^1^ Sichuan Provincial People’s Hospital, Sichuan Academy of Medical Sciences, School of Medicine of University of Electronic Science and Technology of China, Chengdu, Sichuan, China; ^2^ School of Pharmacy, North Sichuan Medical College, Nanchong, Sichuan, China; ^3^ Sichuan Provincial People’s Hospital (Medical Group), Dongli Hospital, Sichuan Academy of Medical Sciences and Sichuan Provincial People’s Hospital, Chengdu, Sichuan, China; ^4^ State Key Laboratory of Quality Research in Chinese Medicine, School of Pharmacy, Macau University of Science and Technology, Taipa, Macau, China

**Keywords:** leonurine, multiple myeloma, monocyte derived dendritic cells, maturation, metabolomics

## Abstract

**Objective:** Leonurine is a bioactive alkaloid compound extracted from Leonurus japonicus Houtt, which potentially has immunomodulatory effects. The immunomodulatory effect and mechanism of leonurine on monocyte derived dendritic cells (moDCs) from healthy donors (HDs) and multiple myeloma (MM) patients were investigated for the first time.

**Methods:** Peripheral blood from HDs and MM patients was isolated for peripheral blood mononuclear cells (PBMCs). The generation of moDCs was conducted by the incubation of monocytes from PBMCs in the medium consisting of RPMI 1640 medium, 2 mmol/L L-glutamine, 5% human serum, 800 U/mL GM-CSF, 500 U/mL IL-4, 100 U/mL penicillin and 0.1 mg/mL streptomycin. During the incubation of 7 days, the cells were administrated with 1 μM leonurine or 1 × PBS as the control group. On the 8th day, cells were harvested. The expression of maturation associated surface markers CD40, CD83, and HLA-DR on moDCs was analyzed by flow cytometry. Moreover, moDCs with or without 1 μM leonurine administration were evaluated by LC-MS/MS for metabolomics which was further analyzed for the potential mechanism of leonurine on moDCs.

**Results:** The proportion of moDCs in the harvested cells was significantly higher in the HD group (*n* = 14) than in the MM patient group (*n* = 11) (*p* = 0.000). Leonurine significantly enhanced the median fluorescence intensity of CD83, HLA-DR and CD40 expression on HD-moDCs (*n* = 14; *p* = 0.042, *p* = 0.013, *p* = 0.084) as well as MM paitent-moDCs (*n* = 11; *p* = 0.020, *p* = 0.006, *p* = 0.025). The metabolomics data showed that in moDCs (HD, *n* = 15), 18 metabolites in the pathway of arachidonic acid metabolism showed significant differences between the leonurine group and the control group (VIP all >1 and *P* all <0.05). To be specific, 6-Keto-PGE1, 8,9-DHET, 11 (R)-HETE, 12-Keto-LTB4, 12-OxoETE, 15 (S)-HETE, 15-Deoxy-Delta12,14-PGJ2, 15-Keto-PGF2a, 20-COOH-LTB4, Lecithin, PGA2, PGB2, PGE2, PGF2a, PGG2, Prostacyclin were significantly upregulated in the leonurine group than in the control group, while Arachidonic Acid and TXB2 were significantly downregulated in the leonurine group than in the control group.

**Conclusion:** Leonurine significantly promotes the maturation of moDCs derived from HDs and MM patients, the mechanism of which is related to arachidonic acid metabolism.

## 1 Introduction

Multiple myeloma (MM) is a hematological malignancy originating from the malignant transformation of plasma cells, the incidence of which is the second highest in hematological malignancies (Pinto et al., 2020). MM patients show significant immunodeficiency. The immunodeficiency of dendritic cells (DCs) is one of the key reasons for the inability to efficiently elicit anticancer specific immune responses. Moreover, cancer can also suppress the normal function of DCs for immune escape. DC is the most important antigen-presenting cell in the immune system, which presents antigen peptides to specific T cell and provides costimulatory signals for T cell activation. Thus, DC plays the important role in initiating anticancer specific immune responses (Tan et al., 2012). However, adverse conditions in the cancer microenvironment can inhibit the activity of DCs by modulating metabolic programs in DCs (Giovanelli et al., 2019). Therefore, enhancing the DC activity in MM patients to effectively elicit the anticancer specific immune response is one of the main directions for cancer immunotherapy today.

Some studies have shown that the maturation and activity of DCs is closely related to the cellular metabolism, including the glucose metabolism and the fatty acid metabolism. When glycogen phosphorylase is inhibited, the activation of DCs and the immune response mediated by DCs can be significantly inhibited (Curtis et al., 2020). Intracellular glycogen metabolism supports the early function of activated DCs, while inhibition of glycogen degradation significantly impedes DC maturation and impairs their ability to initiate lymphocyte activation (Thwe et al., 2019). Inhibitors of fatty acid synthesis or oxidation can significantly inhibit the activation of DCs, which indicates that fatty acid metabolism plays an important role in the activation of DCs (Qiu et al., 2019). Based on the above background, searching for adjuvants (Ho et al., 2018), which can enhance the maturation and activity of DCs by modulating glucose metabolism and fatty acid metabolism is a promising method to efficiently activate anticancer specific immune responses for an improved clinical efficacy.

Leonurine is a bioactive alkaloid compound extracted from Leonurus japonicus Houtt. Recent researches have proven that leonurine can modulate glucose metabolism and fatty acid metabolism, thus performing significantly protective effects in atherosclerotic disease, diabetes, etc. Huang et al. reported that leonurine modulates glucose metabolism *via* the inhibition of advanced glycation end product (AGE) formation, which exhibits potential to prevent diabetes and its complications (Huang et al., 2015). Jiang et al. reported that leonurine can regulate lipid metabolism *via* the promotion of cholesterol efflux and the reduction of cellular lipid accumulation. This results in the effect of leonurine to prevent atherosclerosis (Jiang et al., 2017). Zhu et al. reported that in the db/db mice model, leonurine (200 mg/kg) administered for 3 weeks significantly reduces the fasting blood glucose level and increases the plasma insulin level. Furthermore, leonurine reduces the plasma triacylglycerol concentration and increases the plasma (high-density lipoprotein)-cholesterol concentration (Huang et al., 2012). Therefore, we hypothesis that leonurine is potential to modulate the glucose metabolism and the fatty acid metabolism of DCs so as to increase the maturation and activity of DCs. Thus, in the current study, we investigated for the first time the effect and mechanism of leonurine on DCs derived from healthy donors (HDs) and MM patients.

## 2 Materials and methods

### 2.1 The storage and administration of leonurine

Leonurine was provided by Prof. Yizhun Zhu from School of Pharmacy, Macau University of Science and Technology, China. Leonurine was firstly dissolved in DMSO (Solarbio, China), which was then stored in aliquots at −20°C. The stored leonurine was diluted with 1 × PBS (Solarbio, China), which was then administrated to the cells at the final concentration of 1 μM in the incubation medium.

### 2.2 Donors

Peripheral blood of 14 HDs and 11 MM patients was used for the investigation on moDC maturation by flow cytometry. The 14 HDs included 7 males and 7 females with the age ranging from 24–37 years old. The 11 MM patients included 6 males and 5 females with the age ranging from 47–77 years old. The MM patients all achieved partial remission (PR) with VRD (bortezomib + lenalidomide + dexamethasone) therapeutic regimen. Peripheral blood of 15 HDs was used for the investigation on the metabolomics of moDC. The 15 HDs included 7 males and 8 females with the age ranging from 22–40 years old. The study has been approved by the Ethics Committee of the Sichuan Provincial People’s Hospital. All donors signed the informed consents.

### 2.3 Generating moDCs *in vitro*

20–30 mL peripheral blood from each donor was isolated for peripheral blood mononuclear cells (PBMCs) by density gradient centrifugation. Firstly, peripheral blood was diluted with the equal volume of 1 × PBS. Then, the diluted blood was pipetted over Human Lymphocyte separation medium Ficoll-Paque (GE Lifesciences, United State) with a volume ratio of 4: 3, which was then centrifuged at 400 × g, acceleration 1, deceleration 0 for 30 min. Thereafter, PBMCs were collected as the buffy coat in the tube. PBMCs were divided evenly into 2 groups. Then, PBMCs were incubated for adhesion in the medium consisting of RPMI 1640 medium (Gibco, United State.), 2 mmol/L L-glutamine (Gibco, United State.), 5% human serum (Sigma, United State), 100 U/mL penicillin (Hyclone, China) and 0.1 mg/mL streptomycin (Hyclone, China) for 24 h. Then non-adherent cells were removed while adherent cells were kept for further incubation. The generation of moDCs was conducted by the incubation of monocytes from adherent cells in the medium consisting of RPMI 1640 medium, 2 mmol/L L-glutamine, 5% human serum, 100 U/mL penicillin, 0.1 mg/mL streptomycin, 800 U/mL GM-CSF (Novoprotein, China) and 500 U/mL IL-4 (Novoprotein, China) for 7 days. On the 1st day of incubation, the 2 groups of cells were administrated with or without 1 μM leonurine. On the 8th day, cells were harvested, which were then evaluated by flow cytometry and LC-MS/MS. The morphology of the moDCs was observed and recorded by the inverted microscope (OLYMPUS, Japan).

### 2.4 The expression of surface markers on moDCs by flow cytometry analysis

1 × 10^5^–1 × 10^6^ of the harvested cells were suspended in 100 ul 1 × PBS and stained with APC anti-human CD40 mAb, FITC anti-human CD80 mAb, APC/Cyanine 7 anti-human CD83 mAb, PE anti-human CD86 mAb and Pacific Blue anti-human HLA-DR mAb (all mAbs are from BioLegend, United State). After the cells were incubated with mAbs for 25–30 min at 4°C in darkness, the stained cells were washed for 2 times by 1 × PBS. Then the cells were suspended in 1×PBS for flow cytometry analysis. FlowJo software was used to analyze the data.

**FIGURE 1:**
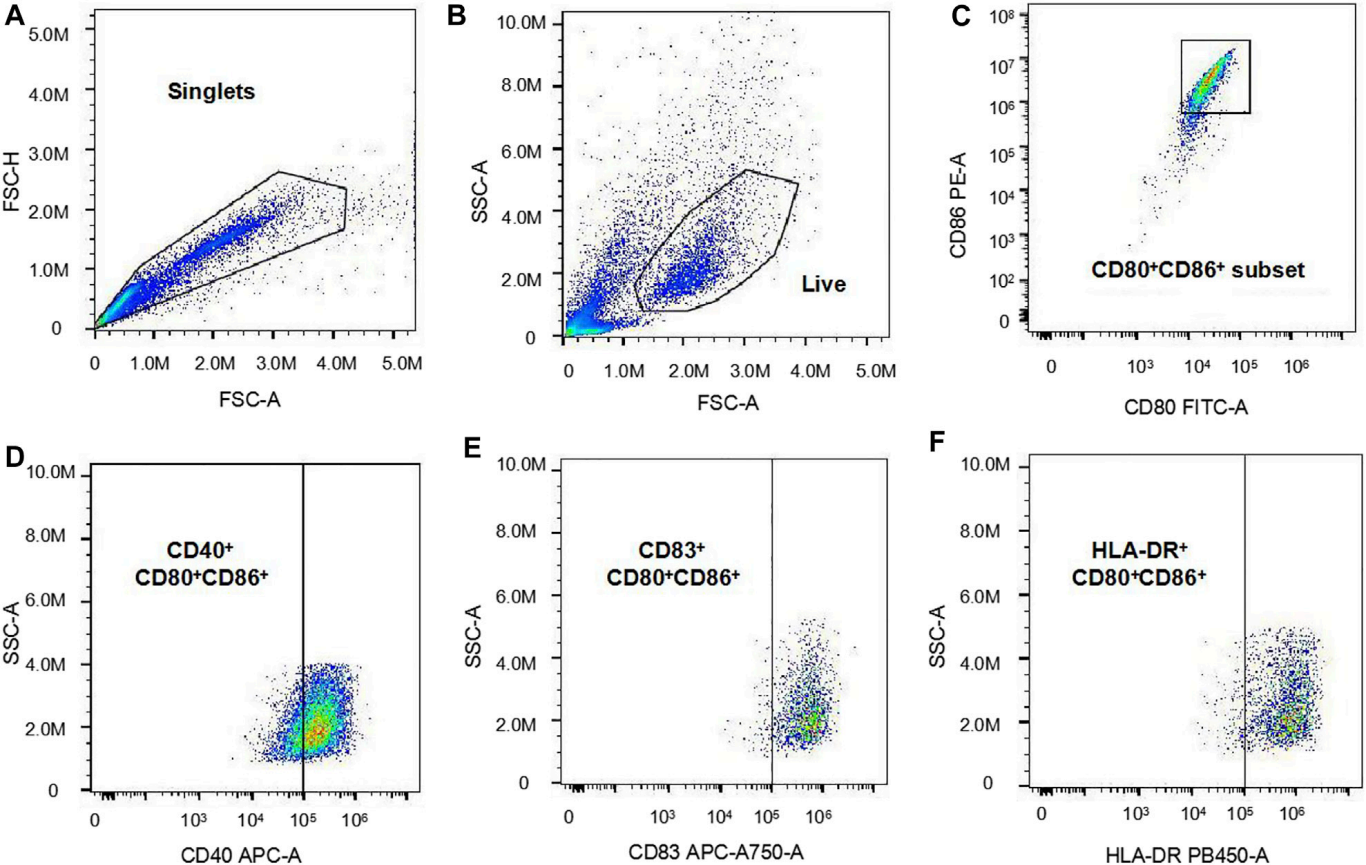
The analysis of the flow cytometry data. **(A)**: The forward scatter area (FSC-A) and the forward scatter height (FSC-H) were used for the selection of single cells. **(B)**: The FSC-A and the side scatter area (SSC-A) were used for the selection of live cells. **(C)**: CD80^+^CD86^+^ cells were selected from the live cells and defined as moDCs **(D–F)**: The MFI of CD40, CD83 and HLA-DR expression on moDCs were analyzed respectively.

In this study, the technique for generating moDCs *in vitro* is a usually used and efficient technique, which can effectively generate moDCs from PBMCs (Chometon et al., 2020). DC expresses high levels of the surface markers CD80 and CD86, which can serve as the definition of DC (Yonggang et al., 2012). Therefore, in our study, the CD80^+^CD86^+^ cell population in the total harvested cells was defined as moDCs. Then, the median fluorescence intensity (MFI) of surface markers CD40, CD83, and HLA-DR expression on moDCs were analyzed respectively. In our study, we used the Cytoflex flow cytometer (Beckman, United State) to analyze the harvested cells. The analysis of the flow cytometry data is displayed as Figure 1. Firstly, the forward scatter area (FSC-A) and the forward scatter height (FSC-H) were used for the selection of single cells from the total cells. Subsequently, the FSC-A and the side scatter area (SSC-A) were used for the selection of live cells from single cells. Then, CD80^+^CD86^+^ cells were selected from the live cells and defined as moDCs. Finally, the MFI of CD40, CD83 and HLA-DR expression on moDCs were analyzed respectively.

### 2.5 Evaluation and analysis of metabolomics

HD-moDCs (*n* = 15) were evaluated for metabolomics. 1 × 10^6^ harvested cells were mixed with 200 μL ultrapure water and vortexed for 30 s. Subsequently, the samples were quickly frozen with liquid nitrogen and then quickly thawed for 3 times. Then, the samples were sonicated for 10 min in ice-water bath. 50 μL of the obtained homogenate was used for the detection of protein concentration. The rest 150 μL homogenate was added with 450 μL methanol (CNW Technologies, Germany) and vortexed for 30 s. The samples were kept at −40°C for 1 h and then centrifuged at 13800 × g for 15 min at 4°C. 550 μL supernatant was transferred into an EP tube and dried in a vacuum concentrator. Then the diluted methanol (methanol: water = 3: 1) with isotopically-labelled internal standard mixture was added into the EP tube and vortexed for 30 s. The samples were sonicated for 10 min in ice-water bath and then centrifuged at 13800 × g for 15 min at 4°C. The obtained supernatant was transferred into a fresh glass vial for analysis.

Liquid chromatography-tandem mass spectrometry (LC-MS/MS) analysis was performed by an UHPLC system (Thermo Fisher Scientific, United State) with a UPLC HSS T3 column (2.1 × 100 mm, 1.8 μm) coupled to Orbitrap Exploris 120 mass spectrometer (Thermo Fisher Scientific, United State). The mobile phase A consisted of 5 mmol/L ammonium acetate (Sigma, United State) and 5 mmol/L acetic acid (Fisher Chemical, United State) in water (pH = 9.75)while the mobile phase B consisted of acetonitrile (CNW Technologies, Germany). The auto-sampler was set at 4°C with the injection volume of 2 μL. The Orbitrap Exploris 120 mass spectrometer was used to acquire MS/MS spectra on information-dependent acquisition (IDA) mode under the control of the acquisition software. In this mode, the acquisition software continuously evaluated the full scan MS spectrum. The ESI source conditions were set as the following: Sheath gas flow rate as 50 Arb, Aux gas flow rate as 15 Arb, Capillary temperature as 320°C, Full MS resolution as 60000, MS/MS resolution as 15000, Collision energy as 10/30/60 in NCE mode, Spray voltage as 3.8 kV (positive) or −3.4 kV (negative), respectively.

The MS raw data was transformed into the mzXML format by ProteoWizard, which was then processed with R package based on XCMS for peak recognition, peak extraction, peak alignment and integration. Then MS2 database (BiotreeDB) was applied in metabolite annotation. The final dataset containing the information of peak number, sample name and normalized peak area was imported into SIMCA16.0.2 software for multivariate analysis to obtain the variable importance in the projection (VIP) values. Further, the differentially expressed metabolites were screened *via* the criteria of VIP >1 and *p* < 0.05. In addition, the Kyoto Encyclopedia of Genes and Genomes (KEGG) database (http://www.genome.jp/kegg/) was used for pathway analysis.

### 2.6 Statistical analysis

All data was expressed as Mean ± Standard Deviation (SD). All *p* values were two-sided and *p* values < 0.05 (*p < 0.05, **p < 0.01 and ***p < 0.001) were defined to be statistically significant. All data was subjected to normality test. The statistical analysis of data was performed by SPSS Statistics25.0 software. The graph and boxplot were produced with GraphPad Prism5.0 software. Independent-sample *t*-test was used for comparing the differences between the HD group and the MM patient group. When the difference between the leonurine group and the control group was analyzed, data which was normally distributed was analyzed with paired-sample *t*-test while data which was not normally distributed was analyzed with paired-sample Wilcoxon signed-rank test.

## 3 Results

To prime T cell activation, DCs present antigen-bound MHC molecules to naïve T cell meanwhile provide naïve T cell with costimulatory molecules including CD40, CD80, and CD86, etc. CD40 upregulates CD80, CD83, CD86, and HLA-DR, which promote DC maturation (Levin et al., 2017). Typical costimulatory molecules CD80 and CD86 are highly expressed on the surface of DCs. The expression level of costimulatory molecules CD40, CD80, and CD86 can reflect the maturation and activity of DCs (Hua et al., 2012). HLA-DR belongs to the MHC II molecular family, which usually binds to exogenous antigens and is recognized by CD4^+^ T lymphocytes. DCs with the higher level of maturation express a higher level of HLA-DR molecule (Saito et al., 2008). CD83 provides the signals of the T cell differentiation, which is a maturation marker for DCs (Cao et al., 2005; Pinho et al., 2014). Consequently, we chose these five surface markers as DC maturation markers in order to clarify the possible immunomodulatory effects of leonurine on DCs. During the incubation procedure, cells were administrated with 1 μM leonurine or 1 × PBS as the control group. Then cells were harvested and observed for the morphology of moDCs. Afterwards, cells were analyzed for the expression of CD40, CD80, CD83, CD86, and HLA-DR by flow cytometry. Furthermore, moDCs with or without 1 μM leonurine administration were evaluated by LC-MS/MS for metabolomics which was further analyzed for the potential mechanism of leonurine on moDCs.

### 3.1 The morphology of moDCs

**FIGURE 2:**
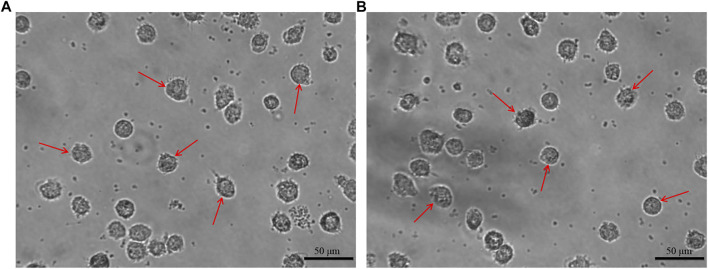
The morphology of moDCs. The moDCs derived from a HD and a MM patient are shown in figure **(A, B)** respectively, which are large, irregular in shape with obvious dendritic protrusions.

After incubation of 7 days, the moDCs were obtained. The morphology of the moDCs was observed and recorded by inverted microscope. The moDCs derived from a HD and a MM patient are shown in Figures 2A, B respectively as the examples. Both Figures 2A, B show that the moDCs are large, irregular in shape with obvious dendritic protrusions on the cell surface.

### 3.2 The comparison between the HD group and the MM group

**FIGURE 3:**
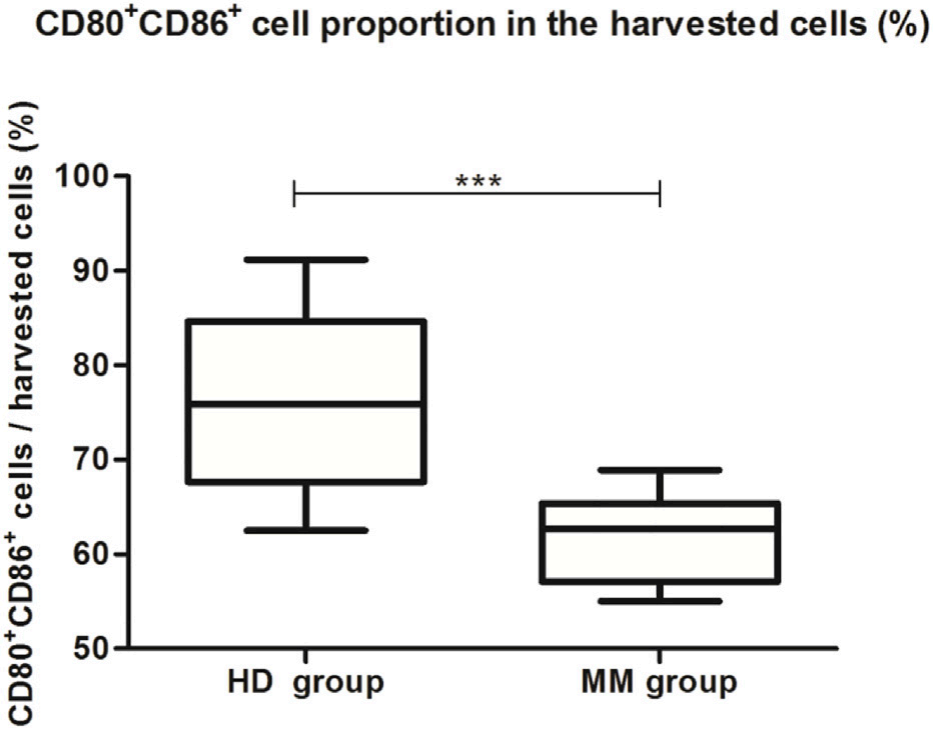
The proportion of moDCs in the harvested cells between the HD group and the MM group. The boxplot shows that the proportion of moDCs in the harvested cells in the HD group was significantly higher than in the MM group (75.55% ± 9.69% vs. 61.39% ± 4.86%, *p* = 0.000, independent-sample *t*-test).

Firstly, we compared the proportion of moDCs (CD80^+^CD86^+^ cells) in the total harvested cells between the HD group and the MM group. The proportion of moDCs in the harvested cells in the HD group was significantly higher than in the MM group (75.55% ± 9.69% vs. 61.39% ± 4.86%, *p* = 0.000, independent-sample *t*-test) (Table 1; Figure 3).

**TABLE 1:** The proportion of moDCs in the harvested cells (%) in the HD group (*n* = 14) and the MM group (*n* = 11).

Group	1	2	3	4	5	6	7	8	9	10	11	12	13	14
HD	85.4	81.4	78.4	68	62.8	91.2	62.5	68.3	68	76.4	88.9	75.4	66.6	84.4
MM	64.1	68.9	65.4	55.1	58.1	63.2	67.2	55	58.5	62.7	57.1			

## 4 The effects of leonurine on the moDCs derived from HDs

### 4.1 The effect of leonurine on the proportion of moDCs in the total harvested cells (%)

**FIGURE 4:**
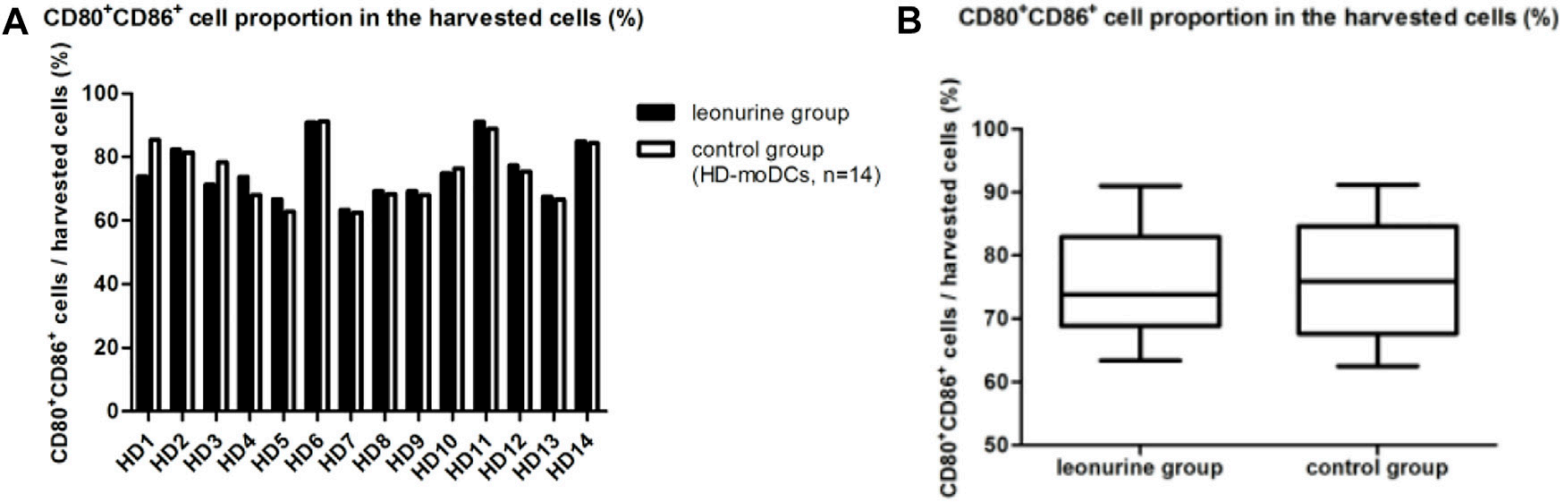
The proportion of moDCs (CD80^+^CD86^+^ cells) in the harvested cells between the leonurine group and the control group (HD-moDCs, *n* = 14). **(A)** The graph shows the proportion of moDCs in the harvested cells between the leonurine group and the control group for each HD. **(B)** The boxplot shows that when moDCs derived from all 14 HDs were analyzed together, there was no statistical difference in the proportion of moDCs in the harvested cells between the leonurine group and the control group (75.45% ± 8.79% vs. 75.55% ± 9.69%, *p* = 0.300, paired-sample Wilcoxon signed-rank test).

When moDCs (CD80^+^ CD86^+^ cells) derived from all 14 HDs were analyzed together, there was no statistical difference in the proportion of moDCs in the harvested cells between the leonurine group and the control group (75.45% ± 8.79% vs. 75.55% ± 9.69%, *p* = 0.300, paired-sample Wilcoxon signed-rank test) (Table 2; Figure 4).

**TABLE 2:** The proportion of moDCs in the harvested cells (%) (HD-moDCs, *n* = 14).

Group	HD1	HD2	HD3	HD4	HD5	HD6	HD7	HD8	HD9	HD10	HD11	HD12	HD13	HD14
Leon	73.9	82.3	71.3	73.7	66.7	90.9	63.4	69.3	69.3	74.8	91	77.3	67.5	84.9
Control	85.4	81.4	78.4	68	62.8	91.2	62.5	68.3	68	76.4	88.9	75.4	66.6	84.4

### 4.2 The effect of leonurine on CD40 expression of moDCs

**FIGURE 5:**
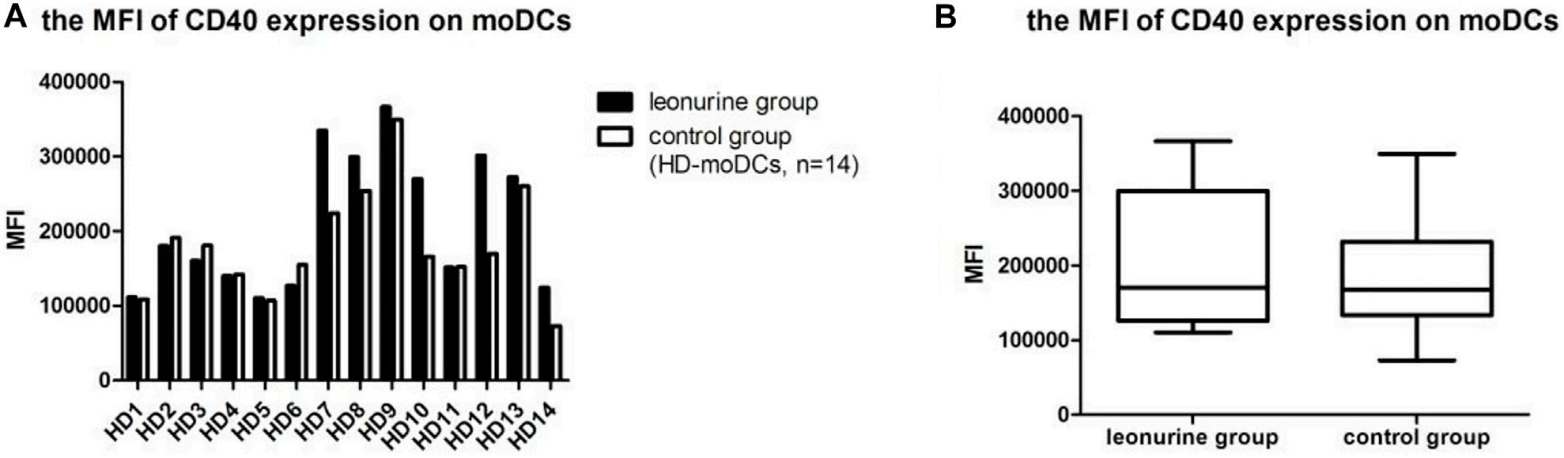
The MFI of CD40 expression on moDCs between the leonurine group and the control group (HD-moDCs, *n* = 14). **(A)** The graph shows the MFI of CD40 expression on moDCs between the leonurine group and the control group for each HD. **(B)** The boxplot shows that when moDCs derived from all 14 HDs were analyzed together, the MFI of CD40 expression on moDCs was significantly higher in the leonurine group than in the control group (2.11 × 10^5^ ± 0.92 × 10^5^ vs. 1.81 × 10^5^ ± 0.72 × 10^5^, *p* = 0.084, paired-sample Wilcoxon signed-rank test).

When moDCs derived from all 14 HDs were analyzed together, the MFI (mean fluorescence intensity) of CD40 expression on moDCs was significantly higher in the leonurine group than in the control group (2.11 × 10^5^ ± 0.92 × 10^5^ vs. 1.81 × 10^5^ ± 0.72 × 10^5^, *p* = 0.084, paired-sample Wilcoxon signed-rank test) (Table 3; Figure 5).

**TABLE 3:** The MFI of CD40 expression on moDCs (×105) (HD-moDCs, n = 14).

Group	HD1	HD2	HD3	HD4	HD5	HD6	HD7	HD8	HD9	HD10	HD11	HD12	HD13	HD14
Leon	1.11	1.80	1.60	1.40	1.11	1.27	3.35	3.00	3.67	2.70	1.52	3.02	2.73	1.24
Control	1.08	1.91	1.81	1.42	1.07	1.55	2.24	2.54	3.49	1.66	1.52	1.70	2.60	0.73

### 4.3 The effect of leonurine on CD83 expression of moDCs

**FIGURE 6:**
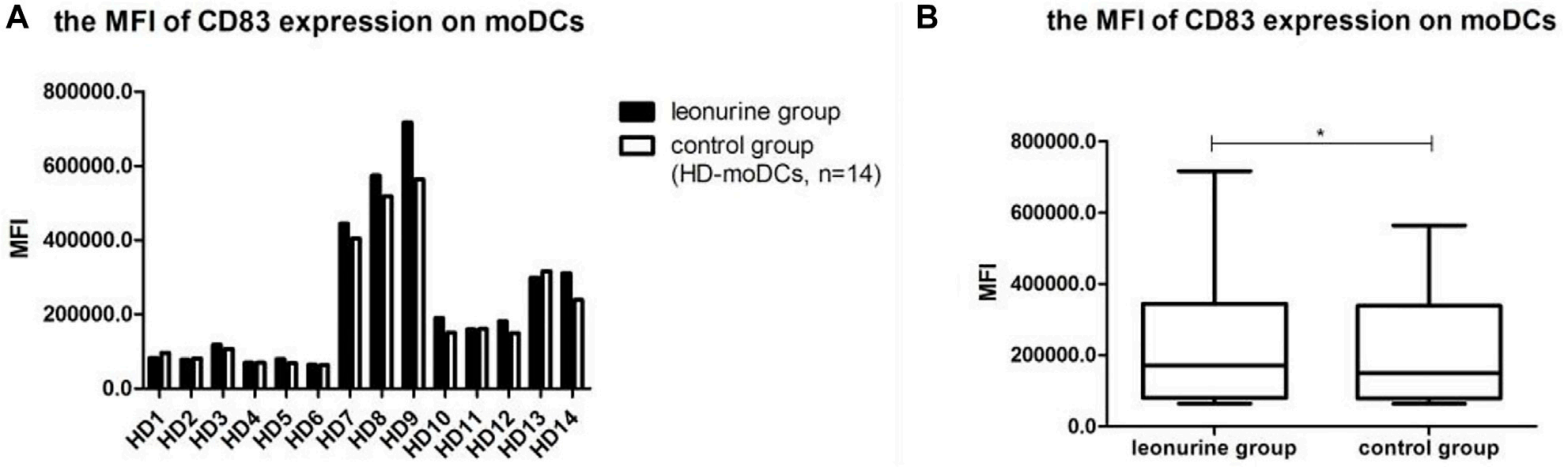
The MFI of CD83 expression on moDCs between the leonurine group and the control group (HD-moDCs, *n* = 14). **(A)** The graph shows the MFI of CD83 expression on moDCs between the leonurine group and the control group for each HD. **(B)** The boxplot shows that when moDCs derived from all 14 HDs were analyzed together, the MFI of CD83 expression on moDCs was significantly higher in the leonurine group than in the control group (2.41 × 10^5^ ± 2.06 × 10^5^ vs. 2.14 × 10^5^ ± 1.71 × 10^5^, *p* = 0.042, paired-sample *t*-test).

When moDCs derived from all 14 HDs were analyzed together, the MFI of CD83 expression on moDCs was significantly higher in the leonurine group than in the control group (2.41 × 10^5^ ± 2.06 × 10^5^ vs. 2.14 × 10^5^ ± 1.71 × 10^5^, *p* = 0.042, paired-sample *t*-test) (Table 4; Figure 6).

**TABLE 4:** The MFI of CD83 expression on moDCs (×105) (HD-moDCs, *n* = 14).

Group	HD1	HD2	HD3	HD4	HD5	HD6	HD7	HD8	HD9	HD10	HD11	HD12	HD13	HD14
Leon	0.82	0.78	1.19	0.70	0.80	0.64	4.44	5.74	7.17	1.89	1.60	1.82	2.99	3.11
Control	0.97	0.81	1.07	0.69	0.69	0.63	4.04	5.18	5.64	1.51	1.61	1.49	3.16	2.39

### 4.4 The effect of leonurine on HLA-DR expression of moDCs

**FIGURE 7:**
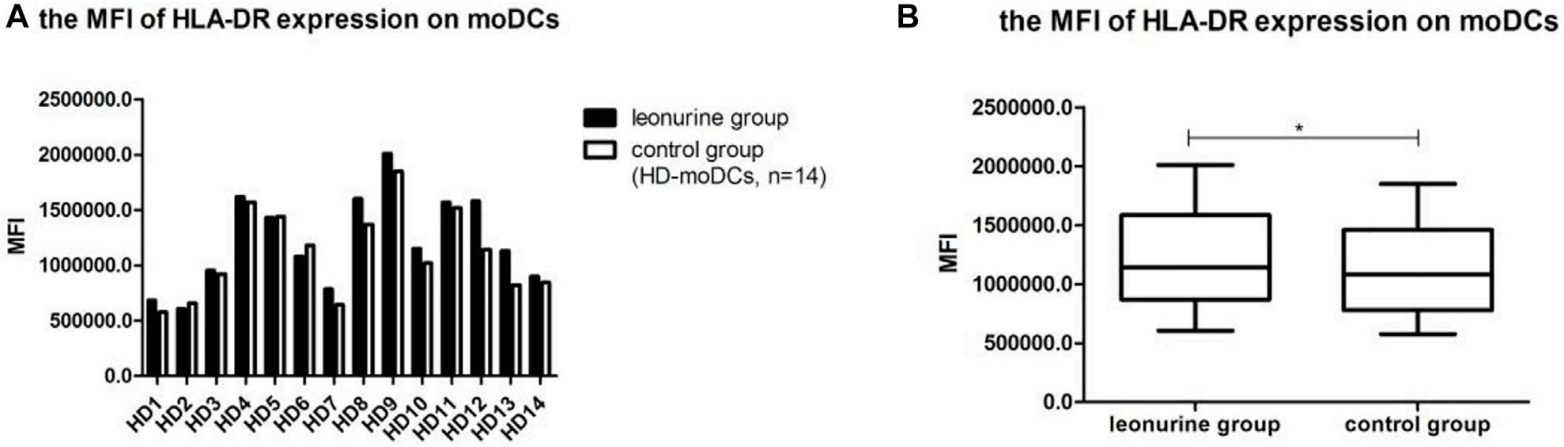
The MFI of HLA-DR expression on moDCs between the leonurine group and the control group (HD-moDCs, *n* = 14). **(A)** The graph shows the MFI of HLA-DR expression on moDCs between the leonurine group and the control group for each HD. **(B)** The boxplot shows that when moDCs derived from all 14 HDs were analyzed together, the MFI of HLA-DR expression on moDCs was significantly higher in the leonurine group than in the control group (12.21 × 10^5^ ± 4.20 × 10^5^ vs. 11.11 × 10^5^ ± 3.94×10^5^, *p* = 0.013, paired-sample *t*-test).

When moDCs derived from all 14 HDs were analyzed together, the MFI of HLA-DR expression on moDCs was significantly higher in the leonurine group than in the control group (12.21 × 10^5^ ± 4.20 × 10^5^ vs. 11.11 × 10^5^ ± 3.94 × 10^5^, *p* = 0.013, paired-sample *t*-test) (Table 5;Figure 7).

**TABLE 5:** The MFI of HLA-DR expression on moDCs (× 105) (HD-moDCs, *n* = 14).

Group	HD1	HD2	HD3	HD4	HD5	HD6	HD7	HD8	HD9	HD10	HD11	HD12	HD13	HD14
Leon	6.82	6.06	9.53	16.2	14.3	10.8	7.85	16.0	20.1	11.5	15.7	15.8	11.3	8.98
Control	5.76	6.58	9.22	15.7	14.4	11.8	6.43	13.7	18.5	10.2	15.2	11.4	8.22	8.45

## 5 The effects of leonurine on the moDCs derived from MM patients

### 5.1 The effect of leonurine on the proportion of moDCs in the total harvested cells (%)

**FIGURE 8:**
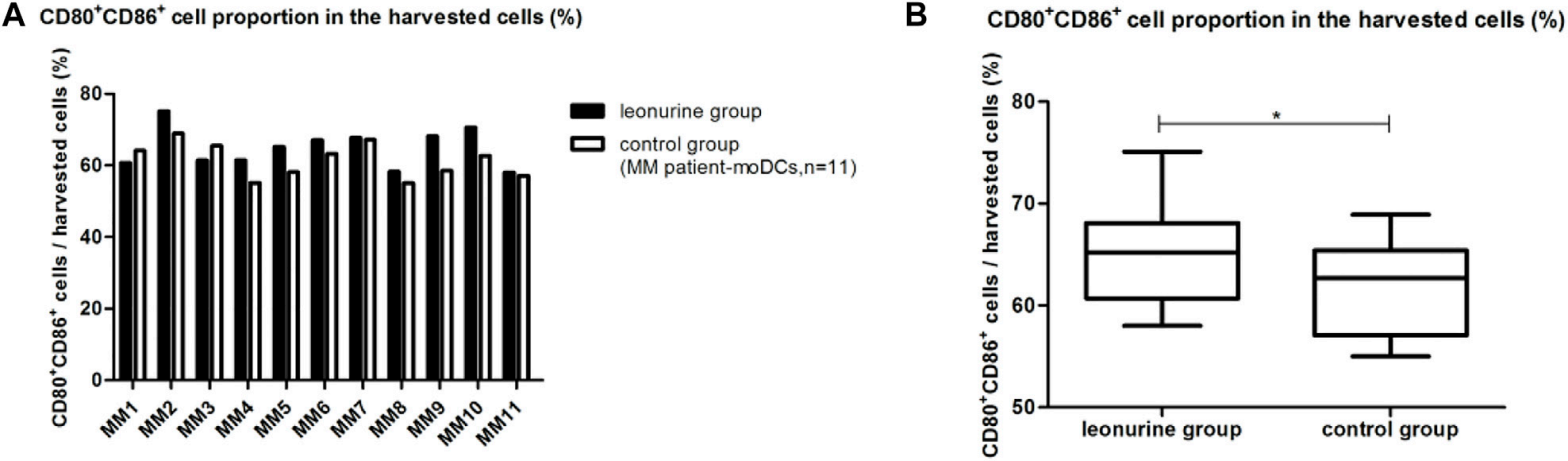
The proportion of moDCs in the harvested cells between the leonurine group and the control group (MM patient-moDCs, *n* = 11). **(A)** The graph shows the proportion of moDCs in the harvested cells between the leonurine group and the control group for each MM patient. **(B)** The boxplot shows that when moDCs derived from all 11 MM patients were analyzed together, the proportion of moDCs in the harvested cells was significantly higher in the leonurine group than in the control group (64.86% ± 5.41% vs. 61.39% ± 4.86%, *p* = 0.029, paired-sample *t*-test).

When moDCs derived from all 11 MM patients were analyzed together, the proportion of moDCs in the harvested cells was significantly higher in the leonurine group than in the control group (64.86% ± 5.41% vs. 61.39% ± 4.86%, *p* = 0.029, paired-sample *t*-test) (Table 6; Figure 8).

**TABLE 6:** The proportion of moDCs in the harvested cells (%) (MM patient-moDCs, *n* = 11).

Group	MM1	MM2	MM3	MM4	MM5	MM6	MM7	MM8	MM9	MM10	MM11
Leon	60.7	75.1	61.4	61.5	65.2	67	67.7	58.2	68.1	70.6	58
Control	64.1	68.9	65.4	55.1	58.1	63.2	67.2	55	58.5	62.7	57.1

### 5.2 The effect of leonurine on CD40 expression of moDCs

**FIGURE 9:**
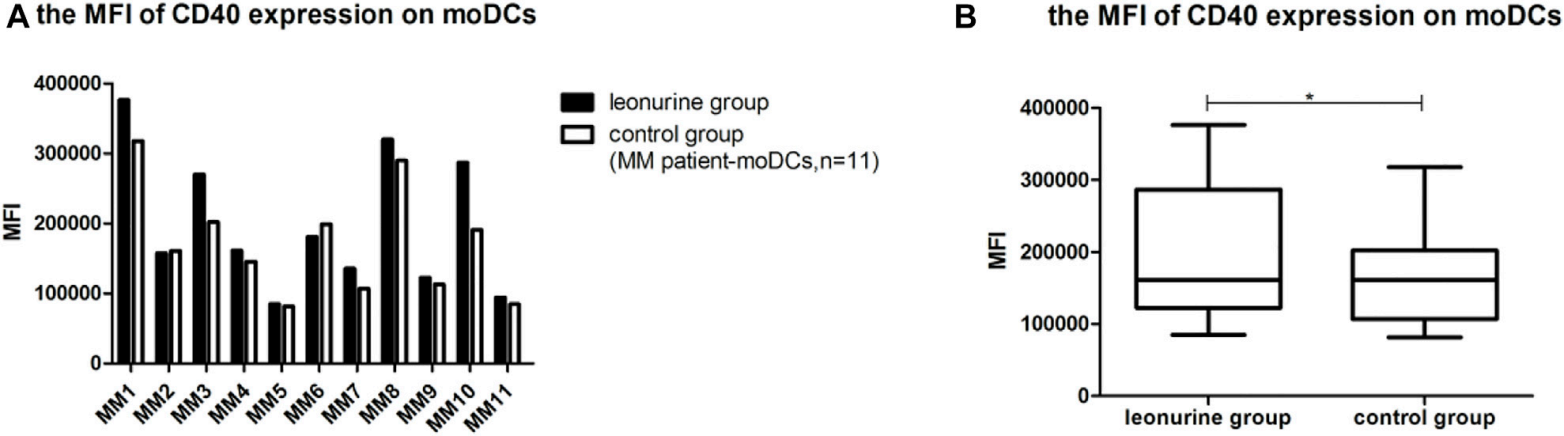
The MFI of CD40 expression on moDCs between the leonurine group and the control group (MM patient-moDCs, *n* = 11). **(A)** The graph shows the MFI of CD40 expression on moDCs between the leonurine group and the control group for each MM patient. **(B)** The boxplot shows that when moDCs derived from all 11 MM patients were analyzed together, the MFI of CD40 expression on moDCs was significantly higher in the leonurine group than in the control group (1.99 × 10^5^ ± 0.98 × 10^5^ vs. 1.72 × 10^5^ ± 0.78×10^5^, *p* = 0.025, paired-sample *t*-test).

When moDCs derived from all 11 MM patients were analyzed together, the MFI of CD40 expression on moDCs was significantly higher in the leonurine group than in the control group (1.99 × 10^5^ ± 0.98 × 10^5^ vs. 1.72 × 10^5^ ± 0.78 × 10^5^, *p* = 0.025, paired-sample *t*-test) (Table 7; Figure 9).

**TABLE 7:** The MFI of CD40 expression on moDCs (× 10^5^) (MM patient-moDCs, *n* = 11).

Group	MM1	MM2	MM3	MM4	MM5	MM6	MM7	MM8	MM9	MM10	MM11
Leon	3.77	1.58	2.70	1.61	0.85	1.81	1.36	3.20	1.22	2.87	0.94
Control	3.18	1.61	2.02	1.45	0.81	1.99	1.07	2.90	1.13	1.91	0.84

### 5.3 The effect of leonurine on CD83 expression of moDCs

**FIGURE 10:**
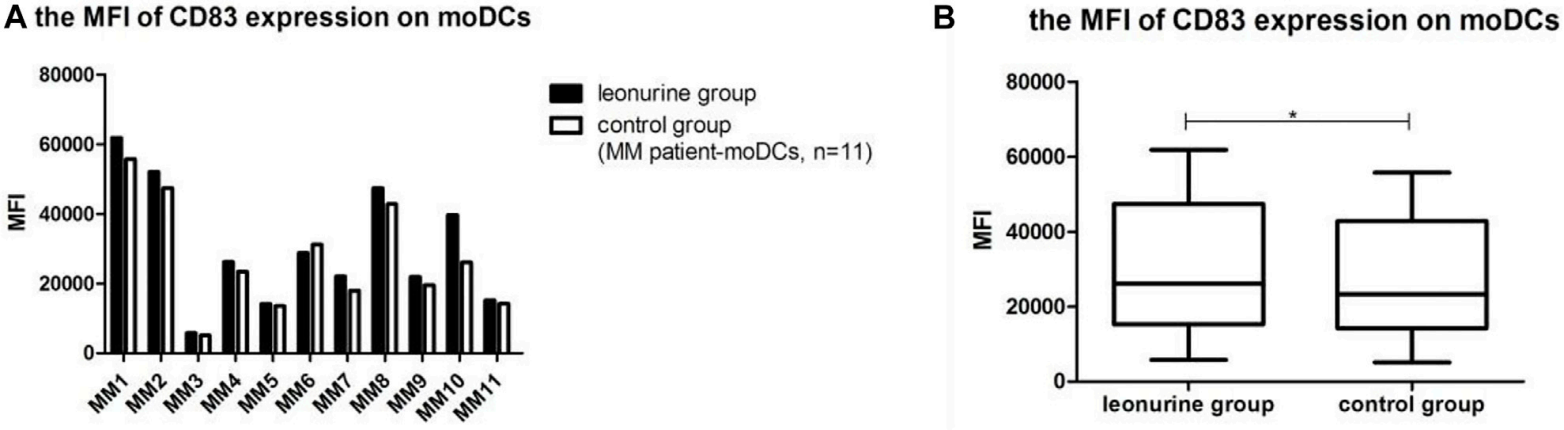
The MFI of CD83 expression on moDCs between the leonurine group and the control group (MM patient-moDCs, *n* = 11). **(A)** The graph shows the MFI of CD83 expression on moDCs between the leonurine group and the control group for each MM patient. **(B)** The boxplot shows that when moDCs derived from all 11 MM patients were analyzed together, the MFI of CD83 expression on moDCs was significantly higher in the leonurine group than in the control group (0.30×10^5^ ± 0.18 × 10^5^ vs. 0.27 × 10^5^ ± 0.16 × 10^5^, *p* = 0.020, paired-sample *t*-test).

When moDCs derived from all 11 MM patients were analyzed together, the MFI of CD83 expression on moDCs in the leonurine group was significantly higher than in the control group (0.30 × 10^5^ ± 0.18 × 10^5^ vs. 0.27 × 10^5^ ± 0.16 × 10^5^, *p* = 0.020, paired-sample *t*-test) (Table 8;Figure 10).

**TABLE 8:** The MFI of CD83 expression on moDCs (× 10^5^) (MM patient-moDCs, *n* = 11).

Group	MM1	MM2	MM3	MM4	MM5	MM6	MM7	MM8	MM9	MM10	MM11
Leon	0.62	0.52	0.06	0.26	0.14	0.29	0.22	0.47	0.22	0.40	0.15
Control	0.56	0.47	0.05	0.23	0.14	0.31	0.18	0.43	0.20	0.26	0.14

### 5.4 The effect of leonurine on HLA-DR expression of moDCs

**FIGURE 11:**
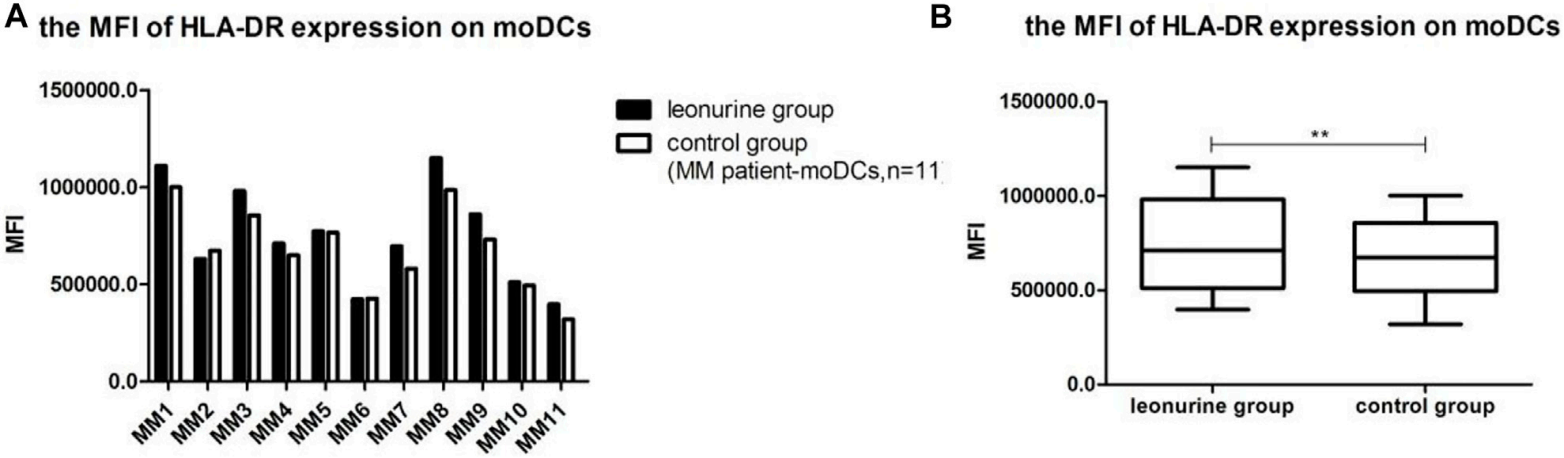
The MFI of HLA-DR expression on moDCs between the leonurine group and the control group (MM patient-moDCs, *n* = 11). **(A)** The graph shows the MFI of HLA-DR expression on moDCs between the leonurine group and the control group for each MM patient. **(B)** The boxplot shows that when moDCs derived from all 11 MM patients were analyzed together, the MFI of HLA-DR expression on moDCs was significantly higher in the leonurine group than in the control group (7.49 × 10^5^ ± 2.58 × 10^5^ vs. 6.80 × 10^5^ ± 2.18 × 10^5^, *p* = 0.006, paired-sample *t*-test).

When moDCs derived from all 11 MM patients were analyzed together, the MFI of HLA-DR expression on moDCs was significantly higher in the leonurine group than in the control group (7.49 × 10^5^ ± 2.58 × 10^5^ vs. 6.80 × 10^5^ ± 2.18 × 10^5^, *p* = 0.006, paired-sample *t*-test) (Table 9;Figure 11).

**TABLE 9:** The MFI of HLA-DR expression on moDCs (× 10^5^) (MM patient-moDCs, *n* = 11).

Group	MM1	MM2	MM3	MM4	MM5	MM6	MM7	MM8	MM9	MM10	MM11
Leon	11.1	6.30	9.81	7.10	7.73	4.23	6.95	11.5	8.61	5.10	3.97
Control	10.0	6.73	8.55	6.49	7.66	4.27	5.80	9.86	7.31	4.94	3.20

## 6 Leonurine regulates on the metabolism of moDCs

**FIGURE 12:**
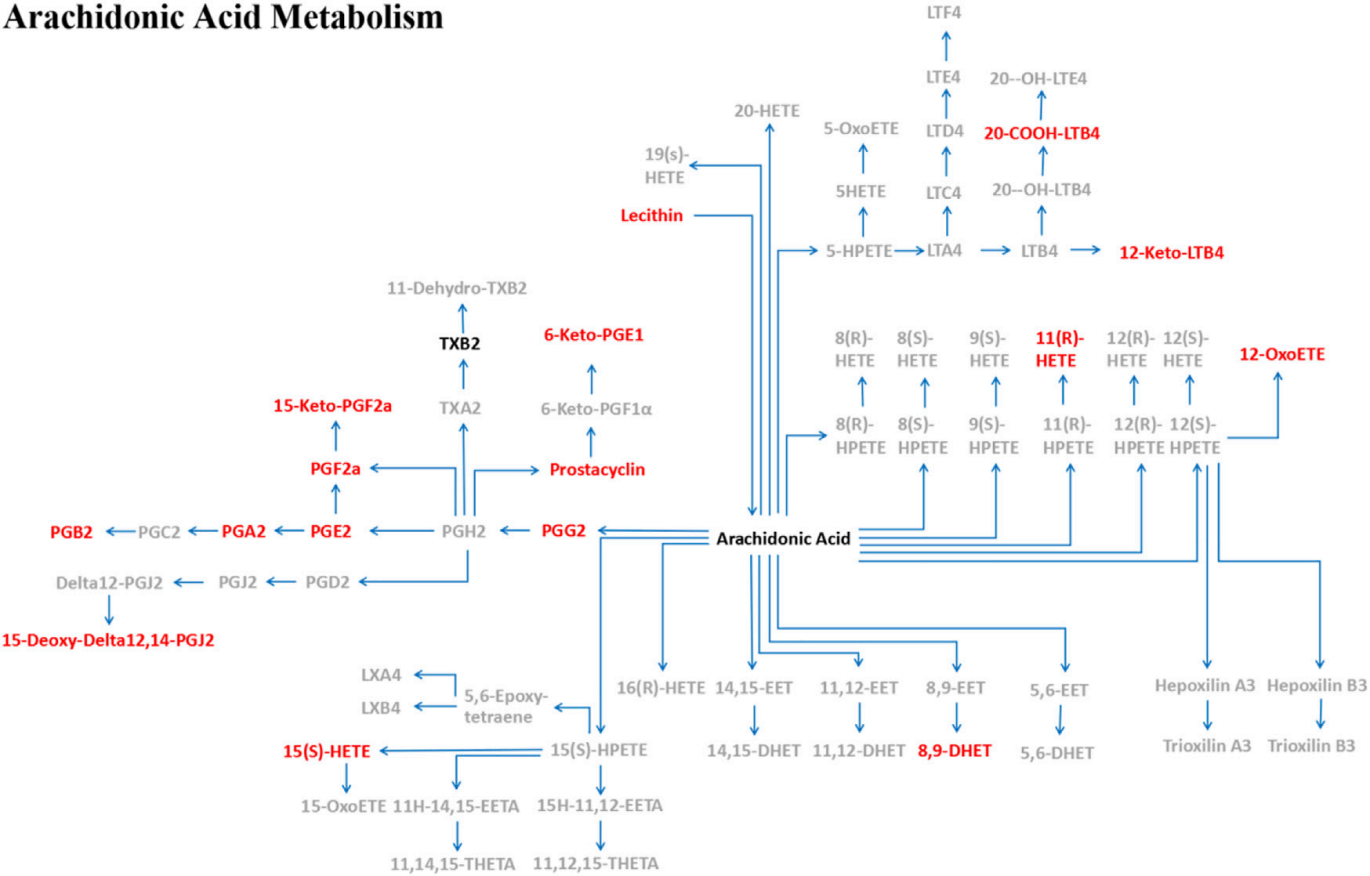
The pathway of arachidonic acid metabolism in moDCs with leonurine administration. After moDCs were administrated with leonurine, 16 metabolites (red) were significantly upregulated while 2 metabolites (black) were significantly downregulated in the pathway of arachidonic acid metabolism. 6-Keto-PGE1: 6-Ketoprostaglandin E1; 8,9-DHET: 8,9-DiHETrE; 12-Keto-LTB4: 12-Keto-leukotriene B4; 12-OxoETE: 12-KETE; 15-Deoxy-Delta12,14-PGJ2: 15-Deoxy-d-12,14-PGJ2; 15-Keto-PGF2a: 15-Keto-prostaglandin F2a; 20-COOH-LTB4: 20-Carboxy-leukotriene B4; Lecithin: PC(22:5 (7Z,10Z,13Z,16Z,19Z)/22:4 (7Z,10Z,13Z,16Z)); PGA2: Prostaglandin A2; PGB2: Prostaglandin B2; PGE2: Prostaglandin E2; PGF2a: Prostaglandin F2a; PGG2: Prostaglandin G2; Prostacyclin: Prostaglandin I2; TXB2: Thromboxane B2.

**FIGURE 13:**
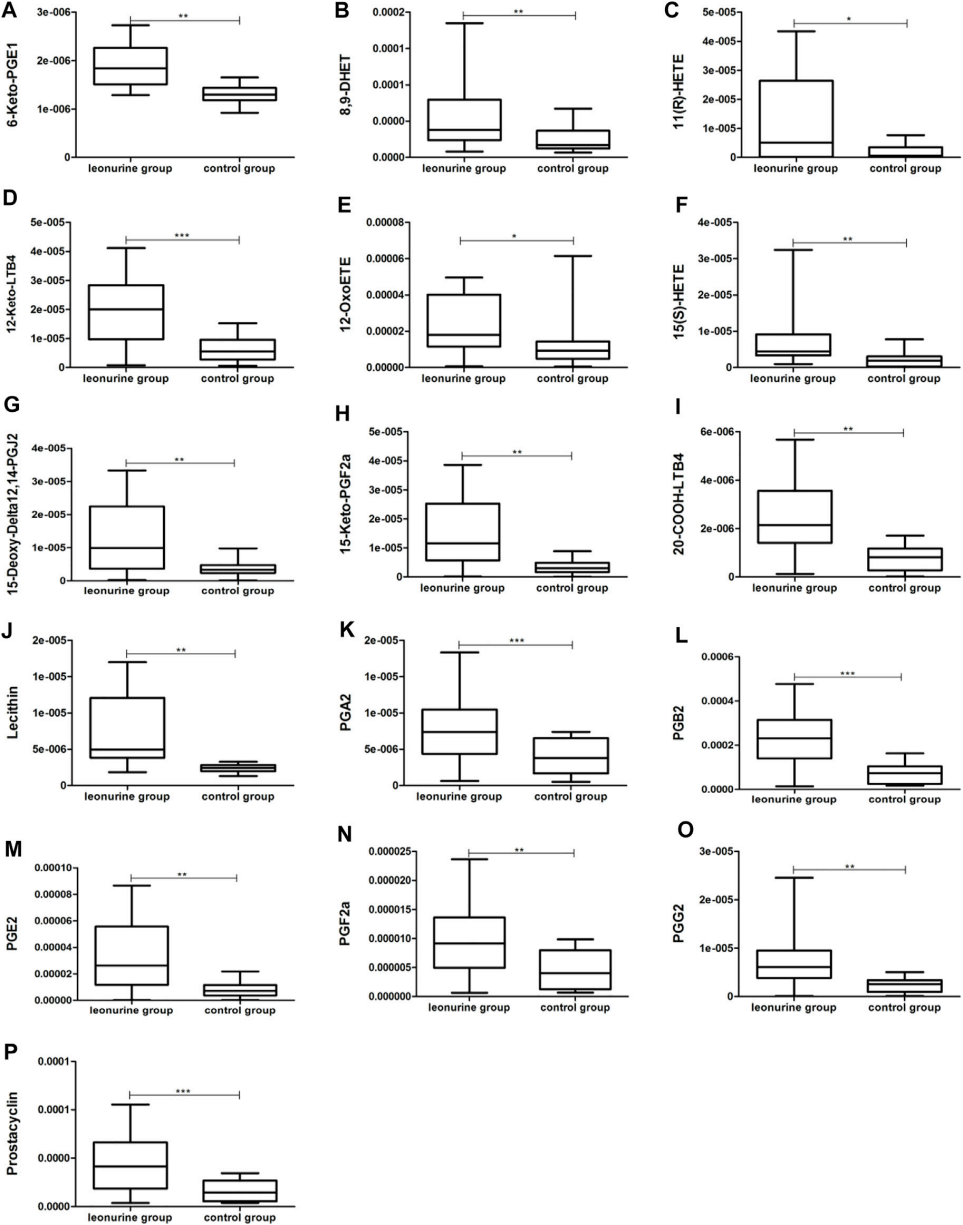
The significantly up-regulated metabolites in moDCs with leonurine administration (HD-moDCs, *n* = 15). 16 metabolites as shown in the above boxplots **(A–P)** were significantly up-regulated in the leonurine group than in the control group.

**FIGURE 14:**
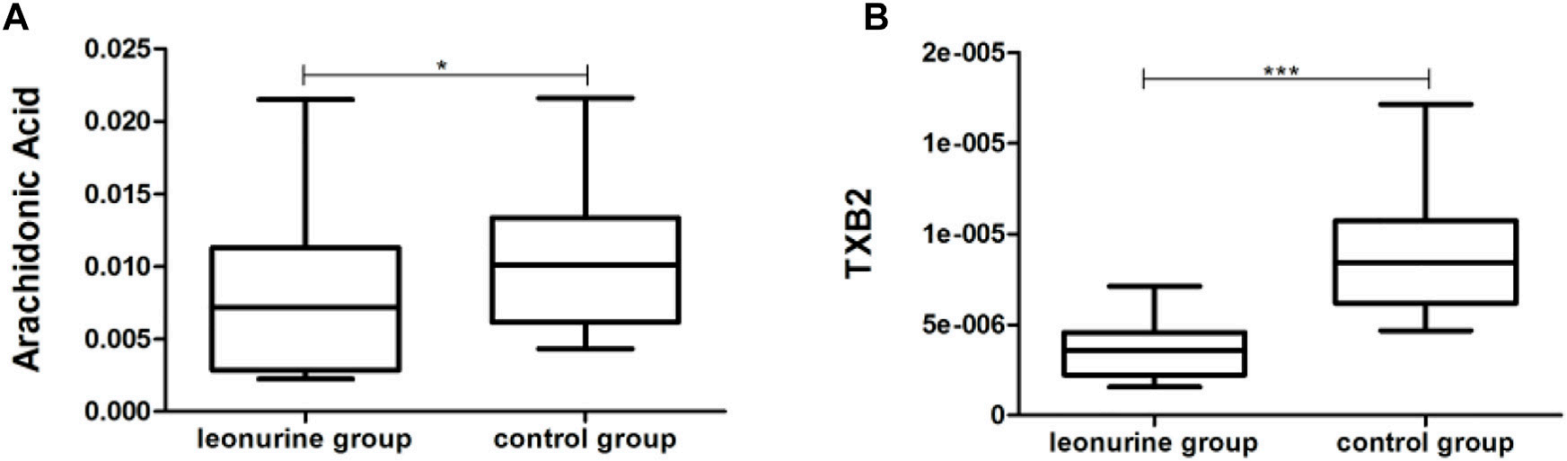
The significantly down-regulated metabolites in moDCs with leonurine administration (HD-moDCs, *n* = 15). 2 metabolites as shown in the above boxplots **(A,B)** were significantly down-regulated in the leonurine group than in the control group.

MoDCs with or without 1 μM leonurine administration (HD-moDCs, n = 15) were evaluated by LC-MS/MS for metabolomics. The metabolomics data showed that in moDCs, 18 metabolites in the pathway of arachidonic acid metabolism were significantly different between the leonurine group and the control group (VIP all >1 and *P* all <0.05; Figure 12). To be specific, 16 metabolites including 6-Keto-PGE1 (Figure 13A), 8,9-DHET (Figure 13B), 11(R)-HETE (Figure 13C), 12-Keto-LTB4 (Figure 13D), 12-OxoETE (Figure 13E), 15(S)-HETE (Figure 13F), 15-Deoxy-Delta12,14-PGJ2 (Figure 13G), 15-Keto-PGF2a (Figure 13H), 20-COOH-LTB4 (Figure 13I), Lecithin (Figure 13J), PGA2 (Figure 13K), PGB2 (Figure 13L), PGE2 (Figure 13M), PGF2a (Figure 13N), PGG2 (Figure 13O) and Prostacyclin (Figure 13P) were significantly up-regulated in the leonurine group than in the control group (Figure 13), while 2 metabolites including Arachidonic Acid (Figure 14A) and TXB2 (Figure 14B) were significantly down-regulated in the leonurine group than in the control group (Figure 14).

## 7 Discussion

MM is a hematological malignancy originating from the malignant transformation of plasma cells, which is still incurable up to date with the second highest incidence in hematological malignancies. MM patients show significant immunodeficiency. Especially, the immunodeficiency of DCs results in the inability of DCs to efficiently elicit anticancer specific immune responses, which leads to the development and recurrence of the disease. Therefore, it is an important therapeutic direction to reverse the immunodeficiency of DCs in MM patients, in order to efficiently activate anticancer specific immune responses and prevent disease progression. In the current study, we for the first time found that leonurine can significantly enhance the maturation and activity of moDCs both in HDs and MM patients. It is shown that leonurine significantly enhances the MFI of CD40, CD83 and HLA-DR expression on HD-moDCs as well as MM patient-moDCs, which indicates that leonurine can promote the maturation and activity of both HD-moDCs and MM patient-moDCs. Moreover, the proportion of moDCs in the total harvested cells is significantly higher in the HD group than in the MM patient group, which also indicates the immunodeficiency of MM patients compared with HDs (Shinde et al., 2018). Therefore, our results show the potential of leonurine as a DC adjuvant to promote the maturation and activity of DCs of MM patients. Leonurine is promising to be applied in DC-based anticancer therapy strategies such as DC vaccine and DC cell-therapy in MM.

As leonurine has been shown in several studies to modulate the glucose metabolism and the fatty acid metabolism, we hypothesis that leonurine is potential to regulate the metabolism of DCs. In the current study, we further confirm that leonurine significantly regulates on the pathway of the arachidonic acid metabolism. The metabolomics data showed that in the pathway of arachidonic acid metabolism, 16 metabolites were significantly upregulated while 2 metabolites were significantly downregulated in moDCs administrated with leonurine. Liu et al. also reported that leonurine can regulate the arachidonic acid metabolism pathway of macrophages by the inhibition of Cyclooxygenase-2, 5-lipoxygenase and microsomal prostaglandin E synthase-1 (Liu et al., 2018). Thus, the mechanism of leonurine is closely related to arachidonic acid metabolism pathway. On the other hand, since the maturation and activity of moDCs is closely related to the cellular metabolism of moDCs including the fatty acid metabolism (Williams and O'Neill, 2018), our study shows the consistent results that arachidonic acid metabolism of moDCs is significantly increased, while the maturation of moDCs is significantly enhanced.

In conclusion, this study indicates for the first time that leonurine significantly promotes the maturation of moDCs derived from both HDs and MM patients, the mechanism of which is related to the regulation on the arachidonic acid metabolism pathway. Leonurine is potential to be applied as a DC adjuvant in DC-based therapeutic strategies for MM patients such as DC vaccine and DC cell therapy.

## Data Availability

The original contributions presented in the study are included in the article/Supplementary Material, further inquiries can be directed to the corresponding authors.
